# Anti-Inflammatory Potential of Complex Extracts of *Ligularia stenocephala* Matsum. & Koidz. and *Secale cereale* L. Sprout in Chronic Gingivitis: In Vitro Investigation and Randomized Clinical Trial

**DOI:** 10.3390/antiox10101586

**Published:** 2021-10-09

**Authors:** Inpyo Hong, Jin-Young Park, Yoo-Hun Noh, Su-Hee Jeon, Jeong-Won Paik, Jung-Seok Lee, Seong-Ho Choi, Jae-Kook Cha

**Affiliations:** 1 Department of Periodontology, Research Institute of Periodontal Regeneration, Yonsei University College of Dentistry, Seoul 03722, Korea; hip0724@gmail.com (I.H.); jypark87@yuhs.ac (J.-Y.P.); ssuhee929@yuhs.ac (S.-H.J.); jpaik@yuhs.ac (J.-W.P.); cooldds@yuhs.ac (J.-S.L.); shchoi726@yuhs.ac (S.-H.C.); 2 Medical Corps of RoKAF 20th Fighter Wing, Seosan 31987, Korea; 3 Innovation Research and Support Center for Dental Science, Yonsei University Dental Hospital, Seoul 03722, Korea; 4 Famenity Co., Ltd., Uiwang-si 16006, Korea; glotesk@gmail.com

**Keywords:** gingivitis, medicinal plants, *Ligularia stenocephala*, *Secale cereale*

## Abstract

Complex extracts of *Ligularia stenocephala* Matsum. & Koidz. (LSE) and *Secale cereale* L. sprout (SCSE) (TEES-10^®^) were prepared. The purposes of the study were to evaluate anti-inflammatory activities of TEES-10^®^ in vitro and to observe resolution of gingivitis in human with oral administration of TEES-10^®^. The effects of TEES-10^®^ on normal periodontal ligament (PDL) cell viability, lipopolysaccharide (LPS) induced PDL cell viability and the changes of inflammatory mediator expression were evaluated in vitro. In the clinical trial, 150 mg of TEES-10^®^ powder containing capsule was administered twice daily to the test group, while the control group administered placebos in a total 100 participants with gingivitis. Probing depth (PD), bleeding on probing (BOP), clinical attachment loss, gingival index (GI) and plaque index (PI) were measured at baseline and 4 weeks. Administering TEES-10^®^ showed significant increase in PDL cell viability compared to administering LSE or SCSE alone. In addition, treating TEES-10^®^ to LPS induced PDL cell significantly increased PDL cell viability compared to control. TEES-10^®^ suppressed expression of NF-κB, p-ERK, ERK, COX-2, c-Fos and p-STAT and promoted expression of PPARγ in LPS induced PDL cells. In the clinical trial, significant improvement of GI and BOP was observed in the test group at 4 weeks. In addition, the number of patients diagnosed with gingivitis was significantly reduced in the test group at 4 weeks. Salivary MMP-8 and MMP-9 was also significantly decreased compared to placebo group. Within the limitations of this study, the TEES-10^®^ would have an anti-inflammatory potential clinically in the chronic gingivitis patients.

## 1. Introduction

Periodontal disease is the result of host immune response against biofilm which induces destruction of alveolar bone and periodontal tissue [1]. In healthy states, various inflammatory cytokines and enzymes mediate normal host response to keep balance from microbial challenge. When pathogenic dysbiosis of biofilm exceed the normal host defense system, tissue destructive enzyme such as matrix metalloproteinase (MMP) and inflammatory cytokines provoke inflammation and destruction of periodontal tissue [2]. Gingivitis is the early stage of periodontal disease, of which inflammation is limited to the gingival tissue [3]. Gingivitis is regarded as the prerequisite of periodontitis which accompanies irreversible alveolar bone loss [4]. Hence, the treatment for periodontal disease can have two different approaches: to remove the biofilm, which is a local factor, or modulate the host response.

Amplified host responses result in the destruction of periodontal tissue through degradation of cell membrane by reactive oxygen species (ROS) from phagocytosis, inactivation of proteolytic enzyme inhibitor and activation of proteolytic enzymes such as MMP [5,6]. In diseased states, the homeostasis between antioxidant and ROS is disrupted and inclined to increase of oxidative stress, whereas oxidative stress decreased after periodontal treatment [7].

Host modulation therapy such as intake of functional foods or antioxidative nutrients like vitamin was suggested to enhance host defense system and down-regulate periodontal inflammation [8,9]. By supplementing nutrition or host modulating medications, inflammatory cytokines are suppressed so that subjects can sustain their homeostasis against the oral microbiome dysbiosis, which are recently considered to be related to numerous systemic diseases [10,11]. In a recent clinical study, combinations of vitamin C, vitamin E, lysozyme and carbazochrome which are known to have antioxidative effect showed significant reduction of gingival inflammation [12].

With increasing interests to host modulation therapy with dietary foods, numerous traditional medicinal plants were tested. Among them, extracts of *Ligularia stenocephala* Matsum. & Koidz. (LSE) and *Secale cereale* L. sprout (SCSE) showed its anti-oxidative and anti-inflammatory activities through in vitro studies [13,14]. In addition, in vivo study with mice model showed that *L. stenocephala* improved inflammatory bowel diseases which is a chronic inflammation [13]. In the present study, we produced complex extracts of *L. stenocephala* and *S. cereale* sprout (TEES-10^®^).

Previous studies about host modulation therapy for periodontal disease had been mostly focused on nutrients and medications [15,16]. However, there has been a lack of approach for host modulation therapy with dietary natural plant extracts such as TEES-10^®^. Thus, the main objectives of this study were to evaluate anti-inflammatory activities of TEES-10^®^ in vitro and to confirm improvement in chronic gingivitis in human with oral administration.

## 2. Materials and Methods

### 2.1. Preparation of TEES-10^®^

TEES-10^®^, a complex extract of LSE and SCSE, was provided by Famenity (Uiwang, South Korea). Leaves of *L. stenocephala* and the sprout of *S. cereale* were selected, crushed and extracted twice with 50% aqueous ethanol then filtered. After second filtration, each filtrate was concentrated until brix was 20–25%. According to the ratio of solid content, dextrin added 1:1 in weight and dried to powder forms. LSE and SCSE were mixed in a ratio of 6:4 in weight to be TEES-10^®^. For clinical trials, 150 mg of TEES-10^®^ powder were filled in hard capsules and used as TEES-10^®^-administered group (test group), while dextrin was filled in the same capsules and used as a placebo (placebo group).

### 2.2. Qualitative Analysis of TEES-10^®^

The chlorogenic acid (ChromaDex Inc., Irvine, CA, USA) and the tricin (Carbosynth Ltd., Compton, UK) as standard samples and the test sample (TEES-10^®^) were precisely weighed and dissolved into 50% aqueous methanol. In addition, thereafter, the test sample (5 μL) and standard samples (5 μL, respectively) were subjected to high performance liquid chromatography (HPLC, Agilent 1260 infinity II; Agilent Technologies, Inc., Waldbronn, Germany) using a Supelco Discovery C18 column (diameter, 4.6 mm; length, 250 mm, Sigma-Aldrich Co., St. Louis, MO, USA) filled with octadecylsilyl silica gel (diameter, 5 μm) at 0.7 mL/min of flow rate. Optimum HPLC separation was achieved at 40 °C. UV wave lengths were 330 nm for chlorogenic acid and 350 nm for tricin. The phosphoric acid (Junsei Chemical Co., Ltd., Tokyo, Japan) was mixed with distilled water and acetonitrile (JT Baker, Phillipsburg, NJ, USA), respectively, and these solvents were used as a mobile phase. The different conditions for each standard were using mobile phases A (0.5% phosphoric acid in water) and B (0.5% phosphoric acid in acetonitrile). The mobile phase condition of chlorogenic acid was as follows: 0–7 min (10% B), 7–27 min (10–30% B), 27–28 min (30–70% B), 28–30 min (70% B), 30–31 min (70–10% B) and 31–40 min (10% B). The mobile phase condition of tricin was as follows: 0–10 min (10–30% B), 10–25 min (30% B), 25–27 min (30–90% B), 27–30 min (90% B), 30–31 min (90–10% B) and 31–35 min (10% B).

### 2.3. In Vitro Investigation

#### 2.3.1. Human PDL Cell Culture

Human periodontal ligament (PDL) cell (CEFOgro™, Seoul, Korea) was cultured according to the manufacturer’s protocol [17]. In brief, the cells were cultured in the growth medium containing 100 U/mL penicillin and 100 µg/mL streptomycin. Cells were cultured in a 5% CO_2_ incubator at 37 °C. The culture medium was replaced every two days and subcultures were performed at 90% confluency. In this experiment, cells were used following 4–6 passages.

#### 2.3.2. Effect of TEES-10^®^ on PDL Cell Viability and LPS Induced Inflammation

LSE alone, SCSE alone and TEES-10^®^, which is a combination of LSE and SCSE, at varying concentrations (25, 50 and 100 μg/mL) was treated to the PDL cells. To evaluate the toxicity on PDL cells, the cells were seeded onto 96-well plates at a density of 8 × 10^3^ cells/well. To compare cell viability, 10 µL of 250 µg/mL, 500 µg/mL and 1000 µg/mL of TEES-10^®^ and, 500 µg/mL and 1000 µg/mL of LSE and SCSE were applied to 90 μL of medium. Cell viability was evaluated after 24 h.

Lipopolysaccharide (LPS) was applied to induce inflammation of the cells [18]. PDL cells were divided into sham control, LPS treated, TEES-10^®^ 50 μg/mL + LPS treated (Tc50) group and TEES-10^®^ 100 μg/mL + LPS treated (Tc100) group. In LPS treated, Tc50 and Tc100 groups, 1 μg/mL of LPS (Sigma-Aldrich Co., St. Louis, MO, USA) was added onto plates to induce inflammation to the PDL cells. Then, 50 and 100 μg/mL TEES-10^®^ were added to each Tc50 and Tc100 groups.

After 24 h of incubation, change of PDL cell viability was observed using commercially available cell viability assay kit (EZ-Cytox Cell viability assay kit, Daeil Lab Service Co., Seoul, Korea). According to manufacturer’s method, tetrazolium salt solution was treated to each well for 2 h. Then, absorbance was measured at 450 nm using microplate reader (Infinite M200 PRO NanoQuant microplate reader, TECAN, Zurich, Switzerland) [19].

#### 2.3.3. Effect of TEES-10^®^ on the Expressions of Inflammatory Mediator

Each PDL cell was seeded onto 12 dishes at a density of 1 × 10^6^ cells/dish and stabilized in a 5% CO_2_ incubator for 24 h. Dishes were divided into four study groups; sham control, LPS treated group, TEES-10^®^ 50 μg/mL + LPS treated (Ti50) group and TEES-10^®^ 100 μg/mL + LPS treated (Ti100) group. In LPS treated group, 1 μg/mL of LPS was treated to the dishes. Ti50 and Ti100 groups were treated with 1 μg/mL of LPS, then 50 and 100 μg/mL of TEES-10^®^ were applied as allocated. The culture medium was removed after 24 h and the cells were washed in phosphate buffered saline. Intracellular protein was dissolved using 1 mM of phenylmethylsulfonylfluoride (PMSF), 1% protease inhibitor cocktail and NP40 cell lysis buffer (Invitrogen; Grand Island, NY, USA) and centrifuged at 13,000 rpm at 4 °C for 5 min. After separating the supernatant, protein concentration was quantified using the Pierce™ BCA protein assay kit (Invitrogen; Grand Island, NY, USA). After 30 μg of protein was electrophoresed using Bolt™ 4–12% Bis-Tris Plus Gels, proteins were transferred onto a nitrocellulose membrane using iBlot^®^ Transfer Stack (Invitrogen; Grand Island, NY, USA) and iBlot^®^ Gel Transfer Device (Invitrogen; Grand Island, NY, USA). Each membrane was blocked at room temperature for 1 h using 5% skim milk (MB cell; Seoul, Korea) and washed three times using 0.1% TBST buffer (TBS in 0.1% Tween20; Biosesang, Gyeonggi-do, Korea). Primary antibodies against NF-κB (1:1000, Cell signaling Technology, Danvers, MA, USA), p-ERK (1:1000, Cell signaling), ERK (1:1000, Cell signaling), COX-2 (1:1000, Abcam; Cambridge, UK), c-Fos (1:1000, Santa Cruz, Dallas, TX, USA), p-STAT1 (1:1000, Santa Cruz), PPARγ (1:1000, Cell signaling) and β-actin (1:1000, Santa Cruz) were incubated with membranes overnight at 4 °C. The membranes were then incubated with the secondary antibody, HRP-conjugated IgG (1:10,000 dilution), at room temperature for 1 h. After washing three times, protein expression was measured using SuperSignal^®^ West Pico Chemiluminescent Substrate (Thermo Fisher Scientific, Waltham, MA, USA) [20].

### 2.4. Double Blinded Randomized Clinical Trial

#### 2.4.1. Study Design and Population

**Figure 1:**
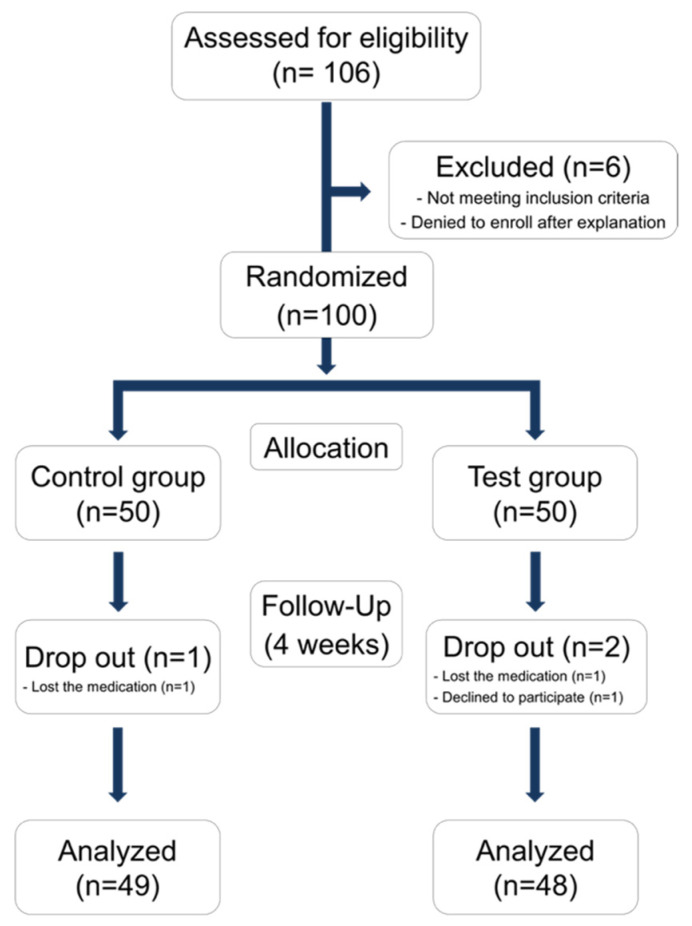
Flow chart of randomized clinical trial.

One hundred voluntary participants were recruited at the clinic of the Department of Periodontology, Yonsei University Dental Hospital. The study protocol was in accordance with the World Medical Association Declaration of Helsinki (Version 2008) and approved by the Institutional Review Board of Yonsei University Dental Hospital (Approval number: 2-2016-0044) and registered with the Clinical Research Information Service (CRIS, Korea, https://cris.nih.go.kr, accessed on 26 January 2017, registration number: KCT0004448). Verbal and written information about the study were given to each participant. Each participant signed an informed consent form before being enrolled in the study. The study design is depicted in Figure 1. Saliva collection, clinical evaluation was done in the first visit. Participants were randomly allocated to test or placebo group, under double blinded condition. In the test group, a TEES-10^®^ capsule was provided twice a day for 4 weeks (300 mg per day). The control group received a placebo, which was delivered in the same shape and color as the TEES-10^®^ capsule for 4 weeks. After 4 weeks, saliva and GCF collection and clinical evaluation was conducted.

#### 2.4.2. Participants

Inclusion criteria were as follows: (1) aged between 19 and 80 years in good general health, (2) having a minimum of 18 teeth, (3) having less than 5 mm of CAL at the first visit and (4) being diagnosed as chronic gingivitis or periodontal health. Criteria for gingivitis were followed to the consensus report of the 2017 World Workshop [21]. Patients exhibiting bleeding on probing (BOP) at more than 10% of the investigated sites were diagnosed as gingivitis. Total 36 sites from the 6 sites (mesiobuccal, midbuccal, distobuccal, mesiolingual, midlingual and distolingual) of 6 representative teeth (#16, 11, 24, 31, 36 and 44) were examined for BOP in this study; thus, participants who had more than four BOP sites were classified into the gingivitis patients.

Exclusion criteria were as follows: (1) not agreed to informed consent, (2) being pregnant or lactating, (3) having a severe systemic disease, (4) taking antiplatelet agents or anticoagulants or having a history of hemorrhage or disease, (5) history of taking an antibiotic more than 3 days within the previous month, (6) having an oral mucosal inflammatory condition (e.g., lichen planus or leukoplakia), or (7) judged as being inappropriate by the clinician for some other reason.

#### 2.4.3. Sample Size Calculation

The sample size was calculated based on previous randomized controlled trials, which evaluated the anti-inflammatory action of nutrient combination using sample size-calculating software (G *Power (Ver. 3.1.9.2), Kiel, Germany) [12]. It was estimated that a sample size of 45 patients in each group would allow for a type II error level of β = 0.20 (80% power) and type I error level of α = 0.05 (5% probability). Considering a dropout rate of 10%, 50 patients were enrolled in each group.

#### 2.4.4. Clinical Evaluation

Six representative teeth (#16, #21, #24, #36, #41 and #44) from each participant were selected for clinical evaluation. Probing depth (PD) and BOP were measured at the mesiobuccal, midbuccal, distobuccal, mesiolingual, midlingual and distolingual sites for each representative tooth. Gingival index (GI) and plaque index (PI) scores were also measured at the mesial, buccal, distal and lingual for each representative tooth [22]. All measurement was done at both baseline and 4 weeks. To compare, mean of GI, PI, PD at each probing site and sum of whole BOP were analyzed.

#### 2.4.5. Salivary Biomarker Evaluation

For collecting whole saliva, cotton roll was used. After soaking the sterilized cotton roll for 5 min, it was put into a saliva collection tube and stored at −80 °C. To measure the concentrations of matrix metalloproteinase (MMP)-8 and MMP-9, the saliva samples were centrifuged at 10,000× *g* at 4 °C for 10 min after thawing, to obtain the supernatant. Respectively, each concentration was measured after dilution at 1:5 and 1:100 with buffer solution. Human MMP-8 ELISA kit (BioVendor-Laboratorní medicína as, Brno, Czech Republic) and MMP-9 Quantikine human MMP-9 Immunoassay ELISA kit (R&D Systems, Minneapolis, MN, USA) were used according to the manufacturer’s instructions. The detection limit of MMP-8 was 0.156 ng/mL to 10 ng/mL and the detection limit of MMP-9 was 0.313 ng/mL to 20 ng/mL. Saliva collection and measurement were conducted at both baseline and 4 weeks.

### 2.5. Statistical Analysis

Statistical analysis was performed using commercially available software (SPSS 24.0, IBM, Armonk, NY, USA); results with *p* < 0.05 were considered statistically significant. All data are presented as mean ± standard error of the mean (SEM). At in vitro test, significant differences were determined using analysis of variance and least-significant differences multi-comparison (LSD) test. At clinical trial, examination variables for effectiveness and safety were treated with an intent-to-treat (ITT) analysis. A paired t-test was used to compare the changes from baseline to 4 weeks between the groups. The incidence rate of gingivitis was calculated for each group and chi-square and McNemar’s test were used for comparative analysis.

## 3. Results

### 3.1. Major Ingredient of TEES-10^®^—Qualitative Analysis of TEES-10^®^

**Figure 2:**
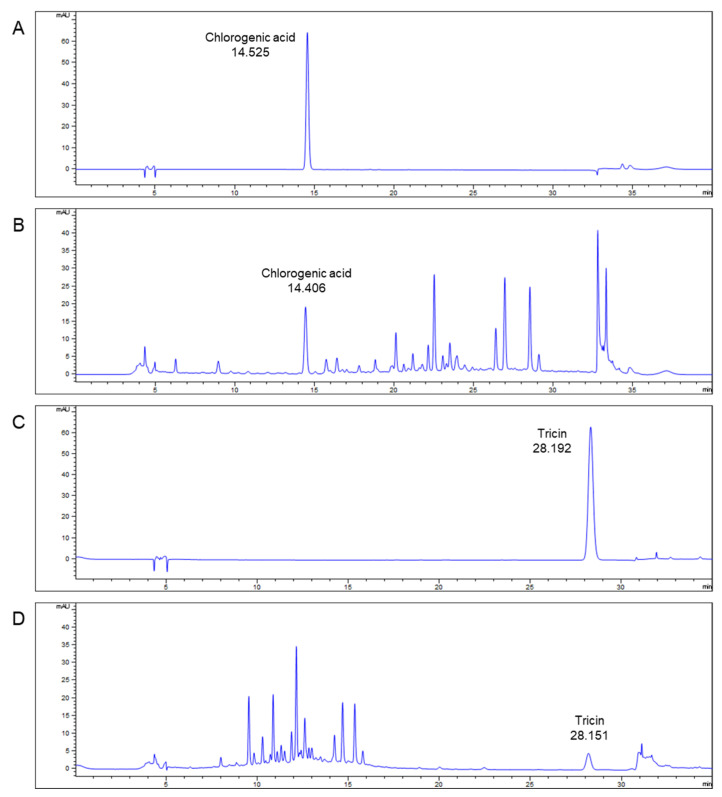
Qualitative analysis of TEES-10^®^ via High Performance Liquid Chromatography. (**A**) and (**B**) were produced under the same mobile phase condition for analysis of chlorogenic acid. (**C**) and (**D**) were produced under the same mobile phase condition for analysis of tricin. The retention times of the standard chlorogenic acid and tricin were 14.525 and 28.192 min, respectively. The retention times of chlorogenic acid and tricin in TEES-10^®^ were 14.406 and 28.151 min, respectively.

The result from the HPLC, the retention time of the chlorogenic acid standard was 14.525 min (Figure 2A), which also was detected in TEES-10^®^ (14.406 min, Figure 2B). The retention time of tricin standard was 28.192 min (Figure 2C), which also was detected in TEES-10^®^ (28.151 min, Figure 2D). Consequently, the major ingredients of the TEES-10^®^ were revealed as chlorogenic acid and tricin.

### 3.2. In Vitro Investigation

#### 3.2.1. Effect of TEES-10^®^ on PDL Cell Viability

**Figure 3:**
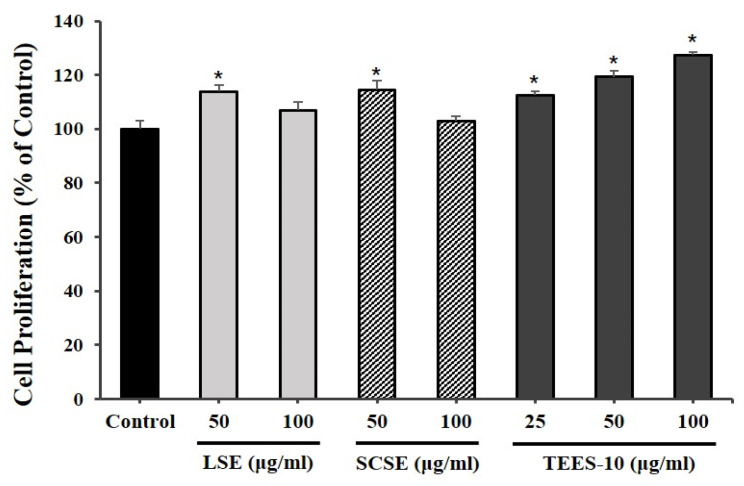
Effects of LSE, SCSE and TEES-10^®^ on periodontal ligament (PDL) cell viability. PDL cells treated with TEES-10^®^ exhibited increased viability in a concentration-dependent manner. Each bar indicates the mean ± SEM. (*n* = 5). * *p* < 0.05 compared with the sham control group. LSE: *L.stenocephala* extract, SCSE: *S. cereale* sprout extract, TEES-10^®^: combination of LSE and SCSE.

LSE concentrations of 50 μg/mL and 100 μg/mL induced cell growth rates of 113.92% and 106.98%, respectively, while the same concentrations of SCSE induced cell growth rates of 114.48% and 103.02%, respectively. However, TEES-10^®^ induced higher cell growth rates of 112.56%, 119.26% and 127.40%, respectively, at concentrations of 25, 50 and 100 μg/mL with a concentration-dependent manner (Figure 3).

#### 3.2.2. Effect of TEES-10^®^ on LPS Induced Inflammation

**Figure 4:**
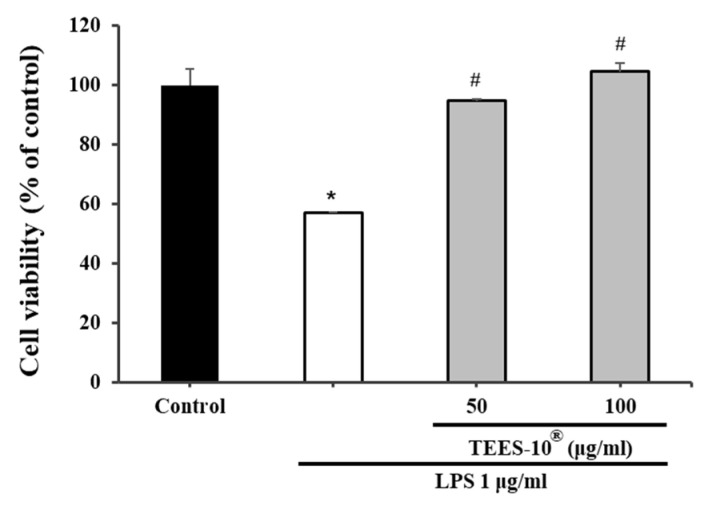
Effects of TEES-10^®^ on the viability of lipopolysaccharide (LPS)-treated PDL cells. Effects of 50 and 100 μg/mL of TEES-10^®^ on LPS-treated PDL cell viability. PDL cells were exposed to 1 μg/mL of LPS. Each bar indicates the mean ± SEM. (*n* = 5). * *p* < 0.05 compared with the sham control group. # *p* < 0.05 compared with the 1 μg/mL LPS-treated group.

After 1 μg/mL of LPS treatment for 24 h, the viability of PDL cells compared to the sham control group was reduced by approximately half. Meanwhile, the viability of the PDL cell was sustained after 1 μg/mL of LPS treatment in conjunction with 50 and 100 μg/mL of TEES-10^®^, respectively (Figure 4).

#### 3.2.3. Effect of TEES-10^®^ on the Expression of Inflammatory Mediators in LPS-Treated PDL Cells

**Figure 5:**
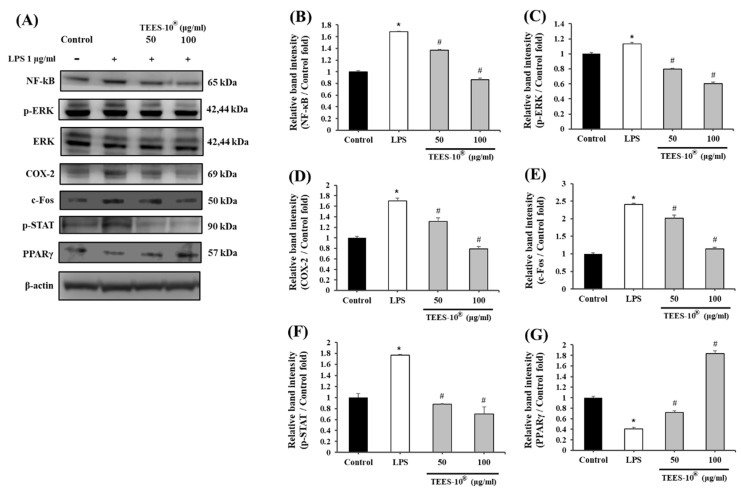
Effects of TEES-10^®^ on NF-κB, p-ERK, ERK, COX-2, c-Fos, p-STAT and PPARγ expression in LPS-treated PDL cells by western blot analysis. (**A**) Protein expression bands of NF-κB, p-ERK, ERK, COX-2, c-Fos, p-STAT and PPARγ. (**B**) Graphs represent relative band intensity of NF-κB compared with β-actin band intensity. (**C**) Graphs represent relative band intensity of p-ERK compared with β-actin band intensity. (**D**) Graphs represent relative band intensity of COX-2 compared with β-actin band intensity. (**E**) Graphs represent relative band intensity of c-Fos compared with β-actin band intensity. (**F**) Graphs represent relative band intensity of p-STAT compared with β-actin band intensity. (**G**) Graphs represent relative band intensity of PPARγ compared with β-actin band intensity. Each bar represents the mean ± SEM (*n* = 3). * *p* < 0.05 compared with the control group. # *p* < 0.05 compared with the 1 μM LPS-treated group.

After 1 μg/mL of LPS treatment for 24 h, the expression of NF-κB, p-ERK and COX-2 increased in the LPS-treated group compared to the control group, which was reduced in a concentration-dependent manner in the TEES-10^®^ treated in conjunction with LPS group compared to the LPS-treated group (Figure 5A–D). The overexpression of NF-κB, p-ERK and COX-2 induced by LPS was significantly reduced by treatment with TEES-10^®^. The expression of c-Fos and p-STAT in the LPS-treated group was more than two times than that of the sham control group, it gradually decreased following administration of 50 μg/mL of TEES-10^®^ and was similar to the sham control group after the administration of 100 μg/mL of TEES-10^®^ (Figure 5A,E,F).

In contrast, the expression of PPAR γ was reduced in the LPS-treated group, but the group treated with 50 μg/mL of TEES-10^®^ in conjunction with LPS showed a slight increase in a concentration-dependent manner compared to the LPS-treated group, while the group treated with 100 μg/mL of TEES-10^®^ showed a notable increase (Figure 5A,G).

### 3.3. Randomized Controlled Study in Human

#### 3.3.1. Demographic Analysis

A total of 106 participants were screened and 100 individuals who met the inclusion and exclusion criteria were randomly assigned to the test (*n* = 50) or control groups (*n* = 50). After random selection, one person withdrew consent and two were excluded because they did not meet the timeline. Finally, 97 individuals (48 and 49 in the test and control groups, respectively) were selected as participants (Figure 1). At the baseline, no statistical differences in age, gender and weight were identified between the groups (Table 1). No abnormal reactions were reported and there was no change of basic lab test in participants during the clinical trial period.

#### 3.3.2. Changes of Clinical Index

Changes of clinical index including GI, PI, BOP and PD are summarized in Table 2 (Table 2). GI score of the TEES-10^®^ intake group was significantly decreased at week 4 (*p* < 0.01) while, that of the placebo group in baseline was sustained until week 4. There was no significant difference between placebo and TEES-10^®^ intake group in baseline; however, at 4 weeks after intake, there was significant difference between placebo and TEES-10^®^ intake group (*p* < 0.05). There was no significant difference between placebo and TEES-10^®^ intake group in PI at either baseline or 4 weeks. There was a slight increase of PI during 4 weeks in both groups.

The BOP sum of the TEES-10^®^ intake group was 8.63 ± 0.77 at baseline; it significantly decreased to 6.44 ± 0.69 at week 4 (*p* < 0.01). At 4 weeks after intake, the BOP sum of control and TEES-10^®^ intake group showed a significant difference between the groups (*p* < 0.01), while there was no significant difference in baseline.

The PD of the TEES-10^®^ intake group was 3.11 ± 0.04 at baseline; it significantly decreased to 3.05 ± 0.04 at week 4 (*p* < 0.05).

#### 3.3.3. Changes in Number of Patients Diagnosed with Gingivitis

The prevalence of gingivitis in each group is summarized in Table 3. The number of patients diagnosed with gingivitis in the TEES-10^®^ intake group showed a statistically significant decrease from 42 subjects to 30 subjects out of total 48 subjects (*p* < 0.01). When comparing between the groups, there was no statistically significant difference at baseline. However, after 4 weeks, the TEES-10^®^ intake group showed a significant decrease in the prevalence of gingivitis compared to the placebo group (*p* < 0.01).

#### 3.3.4. Changes of Salivary Biomarkers for Gingivitis

Changes of matrix metalloproteinase-8 (MMP-8) and matrix metalloproteinase-9 in saliva are summarized in Table 4. TEES-10^®^ intake group showed a statistically significant decrease in MMP-8 levels, from 72.11 ± 14.78 ng/mL to 24.41 ± 4.06 ng/mL, compared to placebo group (*p* < 0.05). MMP-9 level was decreased from 249.93 ± 86.36 ng/mL to 142.97 ± 38.87 ng/mL in the TEES-10^®^ intake group, it showed statistically significant decrease compared to placebo group (*p* < 0.05, Table 4).

No adverse events were reported during the clinical trial period and there was no statistically significant change in weight and blood pressure 4 weeks after administration. The results of clinical laboratory tests such as alanine aminotransferase, aspartate transaminase also showed that the changes were not significant.

## 4. Discussion

In the present study, TEES-10^®^, complex extracts of *L. stenocephala* and *S. cereale* sprout, showed its anti-inflammatory potential to gingivitis at in vitro and clinical levels. The main findings were: (i) TEES-10^®^ significantly increase PDL cell viability in LPS induced inflammatory condition; (ii) TEES-10^®^ significantly decreased expression of inflammatory mediators such as NF-κB, p-ERK, COX-2, c-Fos and p-STAT and increased expression of anti-inflammatory mediators such as PPARγ; (iii) daily intake of TEES-10^®^ would improve gingival health with significantly decreased number of gingivitis patients and reduced MMP-8 concentration.

**Figure 6:**
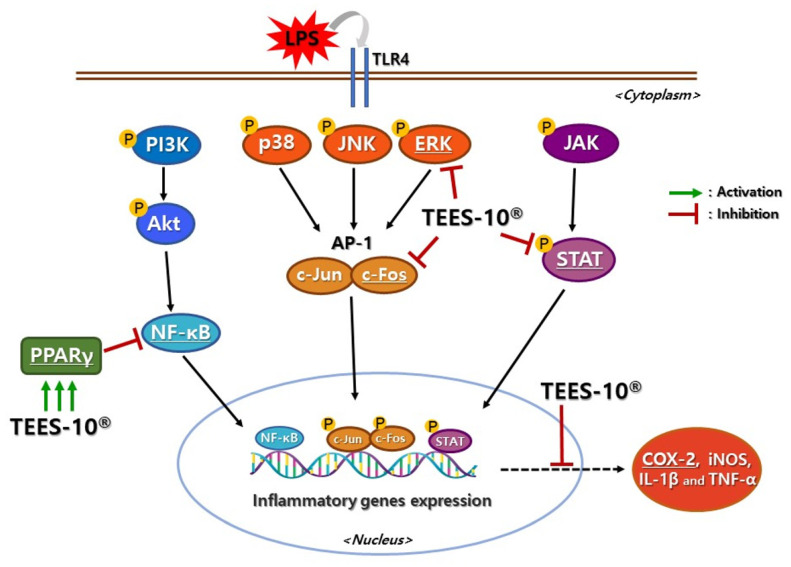
The schematic diagram showing the proposed mechanism of anti-inflammatory effects of TEES-10^®^ in the LPS-induced PDL cell. TEES-10^®^ inhibits production of inflammatory mediators such as NF-κB, p-ERK, c-Fos, p-STAT that mediate inflammatory genes expression in nucleus and TEES-10^®^ decreases COX-2 which is inflammation reaction products. In addition, TEES-10^®^ activates expression of anti-inflammatory mediators such as PPARγ which function inhibition of NF-κB activation. Therefore, TEES-10^®^ could resolute LPS induced inflammation. LPS: Lipopolysaccharide, TLR: Toll-like receptor, PI3K: Phosphatidylinositol-3-phosphate kinase, AKT: Protein kinase B, NF: Nuclear factor, JNK: c-Jun N-terminal kinase, PPAR: Peroxisome-proliferator-activated receptor, ERK: extracellular signal-regulated kinase, AP: Activator protein, JAK: Janus kinase, STAT: signal transducer and activator of transcription, COX: cyclooxygenase, iNOS: inducible nitric oxide synthase, IL: interleukin, TNF: tumor necrosis factor.

There were several studies reported medicinal effects of LSE. By applying LSE, MMP-2, MMP-8 and collagenase, which are related to tissue degradation, were suppressed in cellular level [23]. In addition, NF-kB and ERK, which are key signaling molecules in regulating immune response to infection, were suppressed [13]. In the present study, NF-κB, p-ERK, COX-2, c-Fos and p-STAT were effectively suppressed in inflamed PDL cell by TEES-10^®^ treatment. In 100 μg/mL of TEES-10^®^ treatment, all tested inflammatory mediators except c-Fos showed lower concentration even compared to sham control. It might be regarded that high concentration of TEES-10^®^ facilitates complete resolution of LPS induced inflammation in cellular level (Figure 6).

Extracts of *L. stenocephala* contains phenolic substance represented by flavonoid [13,24]. *S. cereale* sprout also contains phenolic substance and it has been reported that antioxidant effects increase in proportion to the content of phenolic substance [14]. In the present study, TEES-10^®^ consisted of chlorogenic acid, which is a dietary phenolic compound, and tricin, which is dietary flavonoids. Flavonoids and other phenolic compounds are found in many medicinal plants and are in the spotlight as they have beneficial effects, such as antioxidant effects, antibacterial effects, anti-inflammation and immune system promotion [25]. The previous study suggested that phenolic compounds exhibit their antioxidant properties by scavenging radicals, chelating metals and reducing metal ions [26].

Since chronic gingivitis is the result of failure in immune system against dysbiotic change, enhancing the host defense system, which is called host modulation therapy, can reduce gingival inflammation in the same dental plaque accumulated condition [10]. Various phenolic compounds from *L. stenocephala* and *S. cereal* supports the host defense system with its antioxidant ability and reduces periodontal tissue destruction with anti-inflammatory effect such as suppressing nuclear factor κβ, interleukin-6, -10 and -1β [13,27]. The previous review reported various medicinal plants as having antioxidant and phytochemicals which can modulate cellular signaling pathway; also, these medicinal plants can be potential candidates for gingivitis therapeutic [28]. Future studies for host modulation against chronic gingivitis can be conducted with those plants.

Since dietary flavonoids are absorbed through the gastrointestinal tract but are metabolized rapidly, the plasma concentration of flavonoids are usually less than 1 μmol/L [29]. However, numerous studies have shown efficacy of dietary flavonoids and phenolic substances as well, which means that the dietary phenolic substances may also act as cell signaling molecules and induce pathways leading to potential health benefits, rather than the physicochemical mechanisms mentioned above [30]. In this study, through an in vitro experiment, it was confirmed that TEES-10^®^, a combination of LSE and SCSE, induced more effective increase in PDL cell viability proliferation compared to each of LSE or SCSE alone at the same dose. This may imply that there is a synergistic relation between flavonoids in LSE and SCSE. There are cases where signaling-related mechanisms do not show simple dose-dependent effects and in this regard, it can be inferred that the flavonoids of LSE and SCSE may have mutually amplifying signaling relationships.

In the present clinical study, taking a capsule containing 150 mg of TEES-10^®^ powder twice a day showed significant improvement in BOP, GI and gingivitis status. Gingivitis is a condition in which the balance of local factors such as bacterial loads from plaque accumulation and the host immune response is disrupted [4]. Since plaque is an essential factor in the development of gingivitis, mechanical debridement of the plaque by scaling and root planning are regarded as standard and primary periodontal treatment [31]. In the present study, there was no nonsurgical periodontal treatment during the experimental period; nevertheless, gingival inflammation was relieved with only the intake of TEES-10^®^. Therefore, it can be considered that dietary flavonoids from TEES-10^®^ effectively improved host defense of the periodontal tissue.

There were some previous studies that evaluated efficacy of medicinal plants origin phenolic compounds on periodontal disease. The phenolic compounds from medicinal plants showed its effect on periodontal health by inhibiting the inflammatory mediators, as well as the periodontal pathogens [32]. However, in most of the studies, the delivery methods of plant extracts were by direct local delivery systems, such as mouthrinse or gel application [33,34]. Oral administration is efficient in terms of patient convenience, but the difficulty sustaining high concentration of the drug only at the target organ limits its wide application [35]. Despite of these limits of oral administration, TEES-10^®^ showed improvement of inflammation in vitro and clinically. This may imply that anti-inflammatory mechanism of TEES-10^®^ on periodontal tissue is applicable even with low plasma concentration.

In our study, change of MMP-8 and MMP-9 in saliva were measured to confirm improvement of gingivitis. Among various biomarkers, MMP-8 is one of the most sensitive for diagnosing gingivitis [36]. Conventional diagnostic tool for gingivitis is based on manual periodontal probing. Although probing based examination has been used for standard diagnosis criteria of gingivitis, there is the limitation that probing is hard to be standardized due to variety insertion force and angle [37]. In addition, it is difficult to detect changes in gingivitis level which does not demonstrate irreversible alveolar bone loss with periodontal probing. Using biomarkers for diagnosis can supplement the ambiguity of the manual probing and, in particular, it has the advantage of providing objective data to compare and identify the improvements of gingival health as in this study.

Participants of the present study had intake of the capsule containing 150 mg of TEES-10^®^ powder twice daily without any complications. A large number of natural medicinal plant extracts have abundant beneficial phenolic compounds [38]. However, there is a lack of exact information about mechanisms and pharmacodynamics of each compound. Especially, in case of dietary phenolic compounds, miscellaneous factors from habitual diets, demographic character, medication and gut microbiome to genotype affect metabolism of dietary phenolic compounds [39]. Further investigation is needed for determining optimal concentration and formulation of TEES-10^®^.

For future studies, it is needed to confirm exact metabolic mechanism of TEES-10^®^. It is considered that flavonoids take significant role in anti-inflammatory effects of medicinal plants extracts; however, the exact cell signaling pathway is quite imprecise yet. In addition, there are various flavonoids in a single medicinal plant; therefore, the interactions between various flavonoids need to be studied. With a deeper understanding about the mechanisms and interactions of medicinal plants flavonoid, more effective host modulating medicines using natural plants for chronic gingivitis might be developed.

## 5. Conclusions

In summary, TEES-10^®^ showed its anti-inflammatory potential with suppression of inflammatory mediators in cell and improvement of gingivitis in human. Natural derived phenolic substances in TEES-10^®^ are thought to account for anti-inflammatory characteristics. It was noteworthy that even with oral administration, which has limitation of low plasma concentration level, TEES-10^®^ would show an improvement of gingivitis in human. TEES-10^®^ may applied for host modulation therapy for gingivitis with its high anti-inflammatory effects. Future studies to examinppe the mechanisms of medicinal plants derived flavonoid may develop applying medicinal plants to host modulation therapy for chronic gingivitis.

## Figures and Tables

**Table 1:** Demographic table at baseline (mean ± SD).

	Placebo Group	Test Group	*p*-Value
Age (years) Male/Female	32.04 ± 1.80 22/27	32.98 ± 1.97 22/26	*p* > 0.05
			*p* > 0.05
Height (cm)	167.95 ± 1.28	166.70 ± 1.20	*p* > 0.05
Weight (kg)	65.39 ± 2.19	63.72 ± 2.10	*p* > 0.05

**Table 2:** Mean changes in gingival index (GI) scores, plaque index (PI), sum of bleeding on probing (BOP) and probing depth (PD) by groups. Data are presented as the mean ± SE.

	Placebo Group		Test Group	
	At Baseline	At 4 Weeks	At Baseline	At 4 Weeks
GI	0.88 ± 0.06	0.88 ± 0.06	0.88 ± 0.06	0.74 ± 0.07 ^(a) (d)^
PI	0.89 ± 0.11	1.01 ± 0.12	0.96 ± 0.11	1.05 ± 0.13
Sum of BOP	8.51 ± 0.74	8.57 ± 0.69	8.63 ± 0.77	6.44 ± 0.69 ^(b) (d)^
PD (mm)	3.00 ± 0.03	3.10 ± 0.04 ^(c)^	3.11 ± 0.04	3.05 ± 0.04 ^(b) (c)^

^(a)^ *p* < 0.05 compared between groups by two-sample *t*-test. ^(b)^ *p* < 0.01 compared between groups by two-sample *t*-test. ^(c)^ *p* < 0.05 compared within groups by paired *t*-test. ^(d)^ *p* < 0.01 compared within groups by paired *t*-test.

**Table 3:** Changes in number of patients diagnosed with gingivitis by groups. Chi-square test was used for comparing gingivitis prevalence between placebo and TEES-10^®^ intake group in same time period. McNemar test was used for comparing gingivitis incidence between baseline and 4 weeks after TEES-10^®^ or placebo intakes in each group.

	At Baseline		At 4 Weeks		McNemar	
	Placebo	Test	Placebo	Test	Placebo	Test
Gingivitis	40	42	42	30	*p* > 0.05	*p* < 0.01
Healthy	9	6	7	18		
Chi square	*p* > 0.05		*p* < 0.01			

**Table 4:** Mean changes in matrix metalloproteinase-8 (MMP-8) and matrix metalloproteinase-9 (MMP-9) by groups. Data are presented as the mean ± SE.

	Placebo Group (*n* = 49)		Test Group (*n* = 48)	
	At Baseline	At 4 Weeks	At Baseline	At 4 Weeks
MMP-8 (ng/mL)	43.82 ± 11.29	37.85 ± 5.54	72.11 ± 14.78	24.41 ± 4.06 ^(a) (b)^
MMP-9 (ng/mL)	136.58 ± 27.88	194.21 ± 50.54	249.93 ± 86.36	142.97 ± 38.87 ^(a)^

^(a)^ *p* < 0.05 compared between groups by two-sample *t*-test. ^(b)^ *p* < 0.01 compared within groups by paired *t*-test.

## Data Availability

The data is contained within the article.
